# DMSO Concentrations up to 1% are Safe to be Used in the Zebrafish Embryo Developmental Toxicity Assay

**DOI:** 10.3389/ftox.2021.804033

**Published:** 2021-12-21

**Authors:** Jente Hoyberghs, Chloé Bars, Miriam Ayuso, Chris Van Ginneken, Kenn Foubert, Steven Van Cruchten

**Affiliations:** ^1^ Department of Veterinary Sciences, Comparative Perinatal Development, Faculty of Pharmaceutical, Biomedical and Veterinary Sciences, University of Antwerp, Wilrijk, Belgium; ^2^ Department of Pharmaceutical Sciences, Natural Products and Food Research and Analysis (NatuRA), Faculty of Pharmaceutical, Biomedical and Veterinary Sciences, University of Antwerp, Wilrijk, Belgium

**Keywords:** solvent, DMSO, developmental toxicity, embryo, zebrafish, teratogen

## Abstract

Dimethyl sulfoxide (DMSO) is a popular solvent for developmental toxicity testing of chemicals and pharmaceuticals in zebrafish embryos. In general, it is recommended to keep the final DMSO concentration as low as possible for zebrafish embryos, preferably not exceeding 100 μL/L (0.01%). However, higher concentrations of DMSO are often required to dissolve compounds in an aqueous medium. The aim of this study was to determine the highest concentration of DMSO that can be safely used in our standardized Zebrafish Embryo Developmental Toxicity Assay (ZEDTA). In the first part of this study, zebrafish embryos were exposed to different concentrations (0–2%) of DMSO. No increase in lethality or malformations was observed when using DMSO concentrations up to 1%. In a follow-up experiment, we assessed whether compounds that cause no developmental toxicity in the ZEDTA remain negative when dissolved in 1% DMSO, as false positive results due to physiological disturbances by DMSO should be avoided. To this end, zebrafish embryos were exposed to ascorbic acid and hydrochlorothiazide dissolved in 1% DMSO. Negative control groups were also included. No significant increase in malformations or lethality was observed in any of the groups. In conclusion, DMSO concentrations up to 1% can be safely used to dissolve compounds in the ZEDTA.

## 1 Introduction

Zebrafish embryos are gaining interest as an alternative to animal testing for developmental toxicity screening of candidate drugs and chemicals. Zebrafish embryo-based assays are therefore already used for this purpose by different research groups (Brannen et al., 2010; Van den Bulck et al., 2011; Gustafson et al., 2012; Pruvot et al., 2012; Selderslaghs et al., 2012; Lee et al., 2013; Teixidó et al., 2013; Ball et al., 2014; Yamashita et al., 2014; Song et al., 2021) and in our group we refer to this assay as the ZEDTA, i.e. Zebrafish Embryo Developmental Toxicity Assay (Pype et al., 2015; Hoyberghs et al., 2020; Bars et al., 2021). This term will be further used throughout the manuscript for assays that are using zebrafish embryos for developmental toxicity screening of chemicals and pharmaceuticals. The ZEDTA is an *in vitro* test in which the morphological effects of pharmaceuticals and chemicals are assessed in a whole vertebrate organism during the period of organogenesis. Its use during screening has many advantages, as it reduces the use of laboratory animals, it is more cost-effective than the mammalian *in vivo* studies, results are obtained fast due to the short organogenesis period (5.25 h post fertilization (hpf) until 120 hpf) and only a small amount of test compound is needed due to the small size of the embryos. However, many xenobiotics are rather hydrophobic (Weigt et al., 2011; Ball et al., 2014), and therefore organic solvents are needed to solubilize the compounds of interest for exposure experiments in zebrafish embryos (Hutchinson et al., 2006).

Dimethyl sulfoxide (DMSO) is often used to dissolve compounds when using zebrafish embryos for toxicity screening, as it appears to be less toxic in the zebrafish embryo model than other well-known solvents (Kais et al., 2013). High concentrations of DMSO, however, are toxic for zebrafish embryos and larvae (Hallare et al., 2006; Xiong et al., 2017; Huang et al., 2018). Therefore, the Organization for Economic Cooperation and Development (OECD) recommends to keep the final solvent concentration as low as possible, preferably not exceeding 100 μL/L (0.01%), in the (zebra)Fish Embryo Acute Toxicity (zFET) Test (TG236) (Organization for Economic Co-operation and Development (OECD), 2013). However, higher concentrations of DMSO are often required to dissolve compounds in an aqueous medium. Indeed, for teratogenicity screening several research groups report the use of DMSO concentrations that are higher than 0.01% (Brannen et al., 2010; Gustafson et al., 2012; Selderslaghs et al., 2012; Lee et al., 2013; Teixidó et al., 2013; Ball et al., 2014; Yamashita et al., 2014) and this is also true for other types of toxicity assessment, such as developmental neurotoxicity (de Oliveira et al., 2021). However, these DMSO concentrations vary and are scattered in literature, as most laboratories only report the concentration that was needed to dissolve their test compounds. As we recently standardized our ZEDTA (Hoyberghs et al., 2020) with defined exposure window, group size and morphological endpoints to test several compounds, we need to know which concentrations of DMSO are safe to be used in our ZEDTA when dissolving these test compounds.

When reviewing literature, the threshold for morphological abnormalities caused by DMSO appears to vary between 1.0 and 2.5%, depending on the investigated endpoints, exposure duration and developmental stage of the zebrafish (Hallare et al., 2006; Chen et al., 2011; Maes et al., 2012; Xiong et al., 2017; Huang et al., 2018). These upper limits cannot simply be implemented in our standardized ZEDTA, as there are quite some differences in the exposure window and morphological endpoints used in those studies when compared to our standardized protocol (Hallare et al., 2006; Chen et al., 2011; Maes et al., 2012; Xiong et al., 2017; Huang et al., 2018; Hoyberghs et al., 2020). A first difference between our standardized protocol and other studies is the duration of the exposure and stage of the embryo/larva at termination of the study when morphological analysis is undertaken. In our standardized ZEDTA, exposure starts at 5.25 hpf and lasts until 120 hpf (i.e. the period of organogenesis). In several other studies and also in OECD guideline 236, the reference protocol for the zFET test, the exposure started at approximately the same developmental stage, but ended at 96 hpf (Hallare et al., 2004, 2006; Organization for Economic Co-operation and Development (OECD), 2013; Huang et al., 2018; Kim and Lee, 2021). As a result of this shorter exposure period, malformations that occur between 96 hpf and 120 hpf would be missed, and as such the toxic properties of DMSO may be underestimated. In some other studies, the exposure period lasted until after the organogenesis period (Chen et al., 2011; Xiong et al., 2017). In this way, effects that occur after the organogenesis period might overestimate the developmental toxicity of DMSO. Finally, effects of DMSO have also been reported for only a 24 h exposure period and starting from different developmental stages (Maes et al., 2012). This study design is very informative to assess the susceptibility of different developmental stages to DMSO, but as the exposure period is short, effects that occur after a longer exposure period may be missed. A second difference between other studies and our standardized ZEDTA is the list of morphological endpoints that was evaluated, which is often rather limited in other studies. For some studies this can be explained by a focus on other endpoints than gross morphology (Chen et al., 2011; Huang et al., 2018), whereas for others the malformations were not specified and a rather general terminology was used (e.g. abnormal development, crooked body, etc.) (Hallare et al., 2006; Chen et al., 2011; Xiong et al., 2017). As such, the toxic effects of DMSO may be underestimated. Finally, there are also other differences in study design between our standardized ZEDTA and other studies. The number of embryos per group, the number of replicates, the incubation temperature, the number of medium changes and/or the evaluated timepoints, etc. are different from what we use in our standardized ZEDTA (Hoyberghs et al., 2020), and this might also influence the obtained results.

Based on the above, we decided to determine the maximum concentration of DMSO that can safely be used as solvent in our standardized ZEDTA. The results of this study will also benefit the broader scientific community when using this solvent for developmental toxicity testing of xenobiotics in the zebrafish embryo. In a first experiment, we used 2% DMSO as the highest concentration to be tested, as this appeared to be the maximum tolerated DMSO concentration in literature when exposing the zebrafish embryos from 5.25 hpf until 96 hpf. In a second experiment, we evaluated two non-teratogens in combination with the maximum tolerated DMSO concentration of the first experiment, as very recently combined toxic effects of DMSO with chemicals that are non-toxic by themselves have been reported (Kim and Lee, 2021). We opted for ascorbic acid and hydrochlorothiazide, as these compounds were tested in zebrafish embryos at high concentrations in combination with 0.5% DMSO and showed no developmental toxicity (Gustafson et al., 2012). Furthermore, ascorbic acid is water soluble and DMSO is not strictly required, whereas hydrochlorothiazide requires DMSO to be solubilized but at concentrations lower than 1%.

## 2 Materials and Methods

### 2.1 Chemicals and Solutions

Embryo medium was made by dissolving 0.60 g of Instant Ocean® Sea Salt (Blacksburg, VA, United States) and 0.038 g of sodium bicarbonate (Sigma, Diegem, Belgium) in 2 L reverse osmosis (RO) water (pH 7.4 ± 0.3) (Barnstead™ Pacific™ RO Water Purification System, Thermo Scientific™, Waltham, MA, United States ). The MS-222 solution (1 g/L) was made by dissolving methyl ethane sulfonate (i.e. MS-222) (Sigma) in embryo medium, and the pH was adjusted to 7.4 ± 0.3 with 1M NaOH. For the first experiment, DMSO (Sigma) was added to embryo medium to obtain the different DMSO concentrations (0.01%, 0.1%, 0.5%, 1% and 2%). For the second experiment, the following solutions were prepared: embryo medium (medium control), a 1% DMSO (Sigma) solution in embryo medium (solvent control), a 100 µM ascorbic acid (AA) (Sigma) solution in embryo medium, a 100 µM AA (Sigma) solution in embryo medium containing 1% DMSO and a 1,000 µM hydrochlorothiazide (HCT) (Sigma) solution in embryo medium containing 1% DMSO.

### 2.2 Housing and Egg Collection

Experiments were conducted according to our standardized ZEDTA protocol (Hoyberghs et al., 2020). In brief, adult zebrafish (*Danio rerio*) of the AB strain were used as breeding stock. The ratio of males to females was 50/50 and the fish density was <1 fish/L. The 60 L tanks that were used to house the adult fish were filled with reverse osmosis water (Barnstead™ Pacific™ RO Water Purification System, Thermo Scientific™) with Instant Ocean® Sea Salt (Blacksburg) and sodium bicarbonate (Merck, Darmstadt, Germany) to reach a pH of 7.5 ± 0.3 and a conductivity of 500 ± 40 μS/cm. The temperature was set at 28.5 ± 0.3°C, and the tanks were enriched with plastic plants. Fish health and water parameters were checked daily. The limits for ammonia, nitrite and nitrate levels were <0.02 mg/L, <0.3 mg/L and ≤12.5 mg/L, respectively. Adult fish were daily fed with thawed artemia, Daphnia or red, black or white mosquito larvae (alternating; Ruto Frozen Fish food, Montford, The Netherlands). By means of an automated lighting system, fish were exposed to a cycle of 14/10 h light/dark.

For embryo collection, ∼30 adult fish were transferred into a spawning tank the evening before the planned egg collection. To avoid faeces and dirt in the spawning tank as much as possible, fish were fed at the latest at 9 am the morning on the day before collection. To prevent the fish from eating their eggs, the spawning tank was equipped with two nets at the bottom where the eggs could pass through, but the fish could not. On the day of the collection, the fish were allowed to spawn and fertilize eggs for approximately 1 h after the lights turned on. The fish were transferred back to their normal tank, and eggs were collected from the bottom of the spawning tank. To remove the faeces and coagulated eggs, the embryos were washed two times in embryo medium. Then, the embryos were transferred into 48 well plates (Cellstar®, Greiner Bio-One, Frickenhausen, Germany), and only embryos with a normal cell division were selected using an Olympus CKX41 microscope (Olympus U-TV0.5XC-3 lighting; Olympus 4x/0.16 UplanAPO microscope objective) (Olympus Life Science, Shinjuku, Tokyo, Japan). The selected eggs were randomly transferred into new 48 well plates filled with embryo medium and kept at 28.5°C ± 0.3°C in a TIN-IN35 incubator (Phoenix instrument, Garbsen, Germany) with LED strips (LED02102-1, LEDStripXL, Deventer, The Netherlands) attached on the inside. Coagulated and malformed eggs were euthanized with 1 g/L tricaine methane sulfonate (MS-222), pH 7.4 (buffered).

### 2.3 Handling and Exposure of Zebrafish Embryos

#### 2.3.1 First Experiment

The experiment consisted of a medium control group (embryo medium) and five test groups (0.01%, 0.1%, 0.5%, 1% and 2% DMSO). Each experiment (*n* = 20/group) was replicated twice. 48-well plates with a total volume of 300 µL/well were used.

At the latest at 5.25 hpf, the embryos were exposed to the control and test solutions and placed in the incubator (28.5°C ± 0.3°C with a 14/10 h light/dark cycle). To avoid acidification and oxygen deprivation, the embryo medium or test solution was renewed every 48 h (Pype et al., 2015). In addition, the pH of all test solutions was checked prior to exposing the embryos and after an incubation period of 48 h, to make sure a physiological pH was maintained throughout the experiment. A batch of eggs was considered to be valid for experimentation, when a minimum of 80% of all eggs were fertilized and the rate of mortality and malformations of the controls was lower than, or equal to, 10% throughout the experiment (Bars et al., 2021).

#### 2.3.2 Second Experiment

The experiment consisted of a medium control group (embryo medium), a solvent control group (1% DMSO in embryo medium) and three test groups: 1) 100 µM ascorbic acid in embryo medium, 2) 100 µM ascorbic acid in 1% DMSO with embryo medium, and 3) 1,000 µM hydrochlorothiazide in 1% DMSO with embryo medium. The concentrations, 100 µM of ascorbic acid and 1,000 µM of hydrochlorothiazide, were based on Gustafson, et al. (2012) (Gustafson et al., 2012). Each experiment (*n* = 20/group) was replicated twice. 48-well plates with a total volume of 300 µL/well were used. Exposure of embryos to control and test solutions was performed as described in 2.3.1.

### 2.4 Morphological Evaluation

Zebrafish embryos were evaluated for several morphological endpoints (see Table 1) at 5.25, 10, 24, 48, 72, 96 and 120 hpf (Hoyberghs et al., 2020) using an Olympus CKX41 microscope (Olympus Life Science). The endpoints that were evaluated were: coagulation/lethality, no hatching, body parts indistinguishable or unrecognizable, deviations of the tail (curve, elbow and tissue), edema (head, pericard, yolk and yolk extension), blood accumulation (tail, head, heart, yolk and yolk extension), malformation of the pectoral fins (missing or curved), malformation of the cardiovascular system (malformation heart, heartbeat absent, no blood circulation in the tail, disturbed blood circulation in the tail), malformation of the head (deviating shape, deviation ear, deviation mouth, deviation eye), deviating pigmentation, malformation of the yolk and non-detachment of the tail (Hoyberghs et al., 2020). The 5.25 and 10 hpf timepoints were used as a last check-up to replace eggs that coagulated or started to show aberrations in development with spare eggs (also exposed at the latest at 5.25 hpf). From 24 hpf onwards, parameters were checked and scored 0 if they appeared to be normal and 1 if they were malformed. After the last gross morphology scoring at 120 hpf, the larvae were euthanized by means of an overdose of MS-222 (1 g/L in embryo medium) after which they were snap-frozen in liquid nitrogen to ensure death.

**TABLE 1:** General overview of morphological scoring of zebrafish embryos at different developmental stages in the ZEDTA. A detailed list of endpoints can be found in (Hoyberghs et al., 2020).

	Stage (hpf)						
	5.25	10	24	48	72	96	120
Coagulation/lethality	+	+	+	+	+	+	+
Hatching				+	+	+	+
Tail deviation			+	+	+	+	+
Edema			+	+	+	+	+
Blood accumulation			+	+	+	+	+
Malformation of the cardiovascular system			+	+	+	+	+
Malformation of the head			+	+	+	+	+
Malformation of the pectoral fins					+	+	+

Hpf = hours post fertilization.

### 2.5 Statistical Analysis

For the binary scoring data, a Fisher Exact test was used. *p*-values of <0.05 were considered to indicate statistically significant differences. All statistical analyses were performed using GraphPad Prism 8.4.0 or newer versions (GraphPad Software, Inc., San Diego, CA, United States).

## 3 Results

### 3.1 Exposure to a Range of DMSO Concentrations

Both replicates in our first experiment were valid, as ≥80% of all eggs were fertilized and the total number of malformed or dead larvae in the control groups (i.e., embryo solution) was ≤10% at the end of the experiment. The pH of all test solutions remained in a physiological range throughout the experiment (i.e. pH 7.65 ± 0.10) (data not shown). For both replicates, no statistical differences were observed between any of the test groups and the control group (see Table 2 and Table 3). For all test groups, except for the 2% DMSO group, the total number of embryos/larvae that had at least one malformation or were dead at 120 hpf was less than, or equal to, 10% (i.e. 2/20). This cut-off of ≤10% is important, as the highest DMSO concentration will be used as a solvent control in future experiments.

**TABLE 2:** Overview of lethality and malformations in the test groups at 120 hpf in replicate 1. The ratio of affected larvae/total number of larvae is shown for each parameter and each group. For all parameters, except for *coagulation/lethality* and *total ≥ 1 malformations (incl. dead),* this total number of larvae consisted only of larvae that were alive.

Parameter	Control	0.01% DMSO	0.1% DMSO	0.5% DMSO	1% DMSO	2% DMSO
Coagulation/lethality	0/20	1/20	1/20	0/20	0/20	4/20
Tot. ≥1 malf. (incl. dead)	1/20	2/20	2/20	2/20	0/20	6/20
Tot. ≥1 malf. (excl. dead)	1/20	1/19	1/19	2/20	0/20	2/16
BP indistinguishable	0/20	0/19	0/19	0/20	0/20	0/16
BP unrecognizable	0/20	0/19	0/19	0/20	0/20	0/16
No hatching	0/20	0/19	0/19	0/20	0/20	0/16
Elbow tail	0/20	0/19	0/19	0/20	0/20	0/16
Curved tail	0/20	0/19	0/19	2/20	0/20	0/16
Tissue deviation tail	1/20	1/19	0/19	0/20	0/20	2/16
Edema head	0/20	0/19	1/19	0/20	0/20	0/16
Edema pericard	0/20	0/19	1/19	0/20	0/20	0/16
Edema yolk	0/20	0/19	1/19	0/20	0/20	1/16
Edema yolk ext./tail	0/20	0/19	0/19	0/20	0/20	0/16
BA tail	0/20	0/19	0/19	0/20	0/20	0/16
BA head	0/20	0/19	0/19	0/20	0/20	0/16
BA heart	0/20	0/19	0/19	0/20	0/20	0/16
BA yolk	0/20	0/19	0/19	0/20	0/20	0/16
BA yolk extension	0/20	0/19	0/19	0/20	0/20	0/16
Missing fin left	0/20	0/19	0/19	0/20	0/20	0/16
Missing fin right	0/20	0/19	0/19	0/20	0/20	0/16
Curved fin left	0/20	0/19	0/19	0/20	0/20	1/16
Curved fin right	0/20	0/19	0/19	0/20	0/20	0/16
Malformation yolk	0/20	0/19	1/19	0/20	0/20	0/16
Malformation heart	0/20	0/19	0/19	0/20	0/20	0/16
No BC in tail	0/20	0/19	0/19	0/20	0/20	0/16
Disturbed BC in tail	0/20	0/19	0/19	0/20	0/20	1/16
Heartbeat absent	0/20	0/19	0/19	0/20	0/20	0/16
Deviating shape of head	0/20	0/19	0/19	0/20	0/20	0/16
Deviation ear	0/20	0/19	0/19	0/20	0/20	0/16
Deviation mouth	0/20	0/19	1/19	0/20	0/20	0/16
Deviation eye	0/20	0/19	0/19	0/20	0/20	0/16
Deviating pigmentation	0/20	0/19	0/19	0/20	0/20	0/16
Non-detachment tail	0/20	0/19	0/19	0/20	0/20	0/16

BA: blood accumulation, BC: blood circulation, BP: body parts, Tot. ≥1 malf (excl. dead): total number of embryos/larvae that were alive and had at least one malformation. Tot. ≥1 malf (incl. dead): total number of embryos/larvae that had at least one malformation or were dead.

**TABLE 3:** Overview of lethality and malformations in the test groups at 120 hpf in replicate 2. The ratio of affected larvae/total number of larvae is shown for each parameter and each group. For all parameters, except for *coagulation/lethality* and *total ≥ 1 malformations (incl. dead),* this total number of larvae consisted only of larvae that were alive.

Parameter	Control	0.01% DMSO	0.1% DMSO	0.5% DMSO	1% DMSO	2% DMSO
Coagulation/lethality	0/20	0/20	0/20	0/20	1/20	1/20
Tot. ≥1 malf. (incl. dead)	2/20	0/20	1/20	1/20	1/20	5/20
Tot. ≥1 malf. (excl. dead)	2/20	0/20	1/20	1/20	0/19	4/19
BP indistinguishable	0/20	0/20	0/20	0/20	0/19	0/19
BP unrecognizable	0/20	0/20	0/20	0/20	0/19	0/19
No hatching	0/20	0/20	0/20	0/20	0/19	0/19
Elbow tail	0/20	0/20	0/20	0/20	0/19	0/19
Curved tail	1/20	0/20	0/20	0/20	0/19	0/19
Tissue deviation tail	1/20	0/20	1/20	1/20	0/19	4/19
Edema head	0/20	0/20	0/20	0/20	0/19	1/19
Edema pericard	0/20	0/20	0/20	0/20	0/19	1/19
Edema yolk	0/20	0/20	0/20	0/20	0/19	1/19
Edema yolk ext./tail	0/20	0/20	0/20	0/20	0/19	0/19
BA tail	0/20	0/20	0/20	0/20	0/19	0/19
BA head	0/20	0/20	0/20	0/20	0/19	0/19
BA heart	0/20	0/20	0/20	0/20	0/19	0/19
BA yolk	0/20	0/20	0/20	0/20	0/19	0/19
BA yolk extension	0/20	0/20	0/20	0/20	0/19	0/19
Missing fin left	0/20	0/20	0/20	0/20	0/19	0/19
Missing fin right	0/20	0/20	0/20	0/20	0/19	0/19
Curved fin left	0/20	0/20	0/20	0/20	0/19	0/19
Curved fin right	0/20	0/20	0/20	0/20	0/19	0/19
Malformation yolk	0/20	0/20	0/20	0/20	0/19	1/19
Malformation heart	0/20	0/20	0/20	0/20	0/19	0/19
No BC in tail	0/20	0/20	0/20	0/20	0/19	1/19
Disturbed BC in tail	0/20	0/20	0/20	0/20	0/19	0/19
Heartbeat absent	0/20	0/20	0/20	0/20	0/19	0/19
Deviating shape of head	0/20	0/20	0/20	0/20	0/19	1/19
Deviation ear	0/20	0/20	0/20	0/20	0/19	0/19
Deviation mouth	0/20	0/20	0/20	0/20	0/19	0/19
Deviation eye	0/20	0/20	0/20	0/20	0/19	1/19
Deviating pigmentation	0/20	0/20	0/20	0/20	0/19	0/19
Non-detachment tail	0/20	0/20	0/20	0/20	0/19	0/19

BA: blood accumulation, BC: blood circulation, BP: body parts, Tot. ≥1 malf (excl. dead): total number of embryos/larvae that were alive and had at least one malformation. Tot. ≥1 malf (incl. dead): total number of embryos/larvae that had at least one malformation or were dead.

**FIGURE 1:**
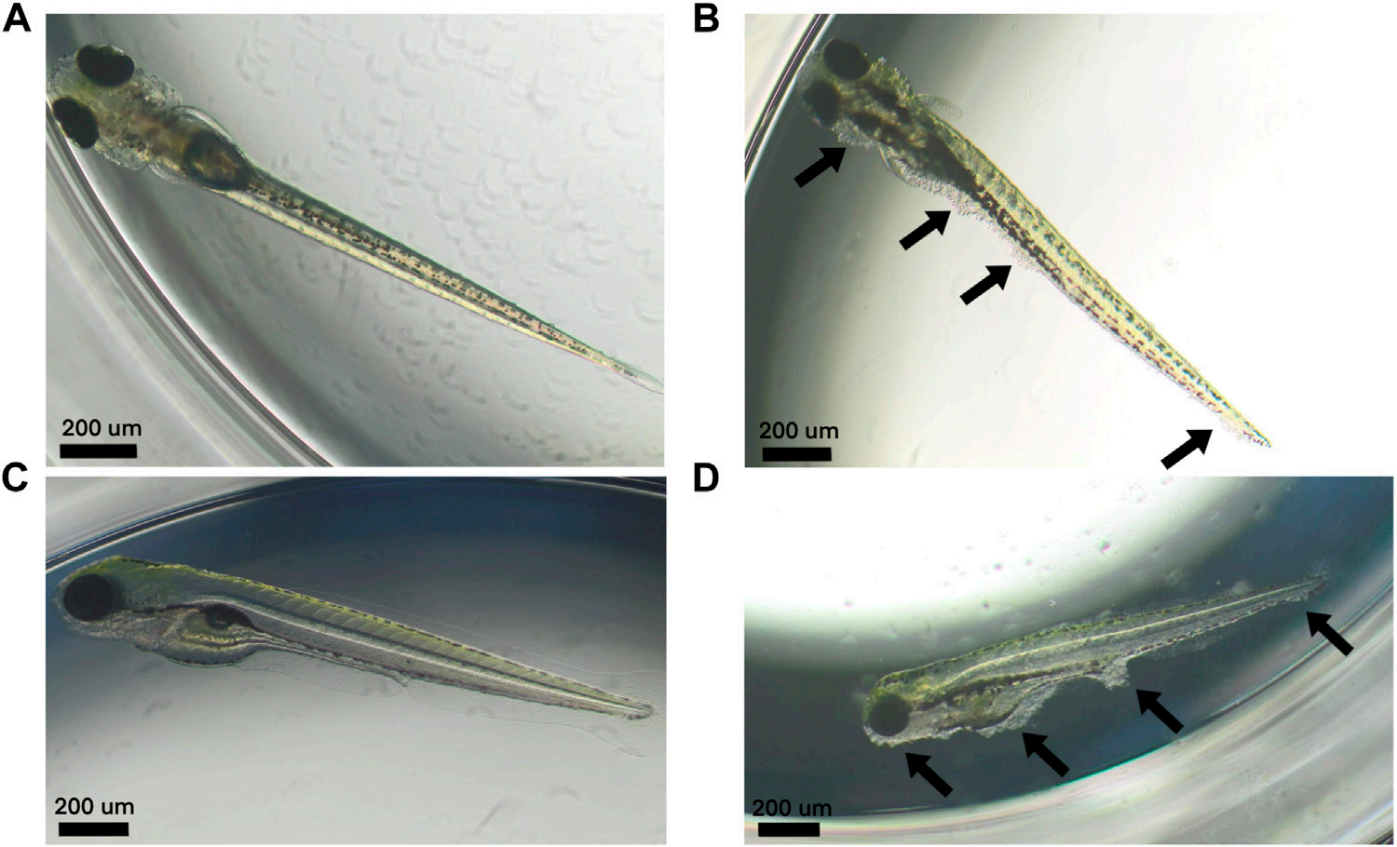
Larvae at 120 hpf. **(A–C)** Normally developed control larvae. **(B–D)** Larvae treated with 2% DMSO that developed tissue deviations of the tail and the body (i.e., areas of cell death) (arrows).

In the 2% DMSO group, a total of six out of 20 larvae (30%) in the first replicate (see Table 2) and 5 out of 20 larvae (25%) in the second replicate (see Table 3) had at least one malformation or were dead. In the first replicate, four of these larvae (20%) were dead, while there was only one larva (5%) in the second replicate. The malformations that were observed in the 2% DMSO group of the first replicate were: tissue deviation of the tail and of the body, yolk edema, curved fin and disturbed blood circulation in the tail. In the second replicate, tissue deviation of the tail and the body, yolk edema, pericardial edema, head edema, malformation of the yolk, no blood circulation in the tail, deviating shape of the head and deviation of the eye were observed. In both replicates, tissue deviation of the tail or/and the body, which was observed as cell death in these areas, showed to be the most prominent malformation (see Figure 1) and was present in all of the alive, malformed larvae. As such, the 2% DMSO group has more than 10% malformed and/or dead larvae, which means that using 2% DMSO as a solvent control group makes the experiment invalid, and therefore cannot be used.

**FIGURE 2:**
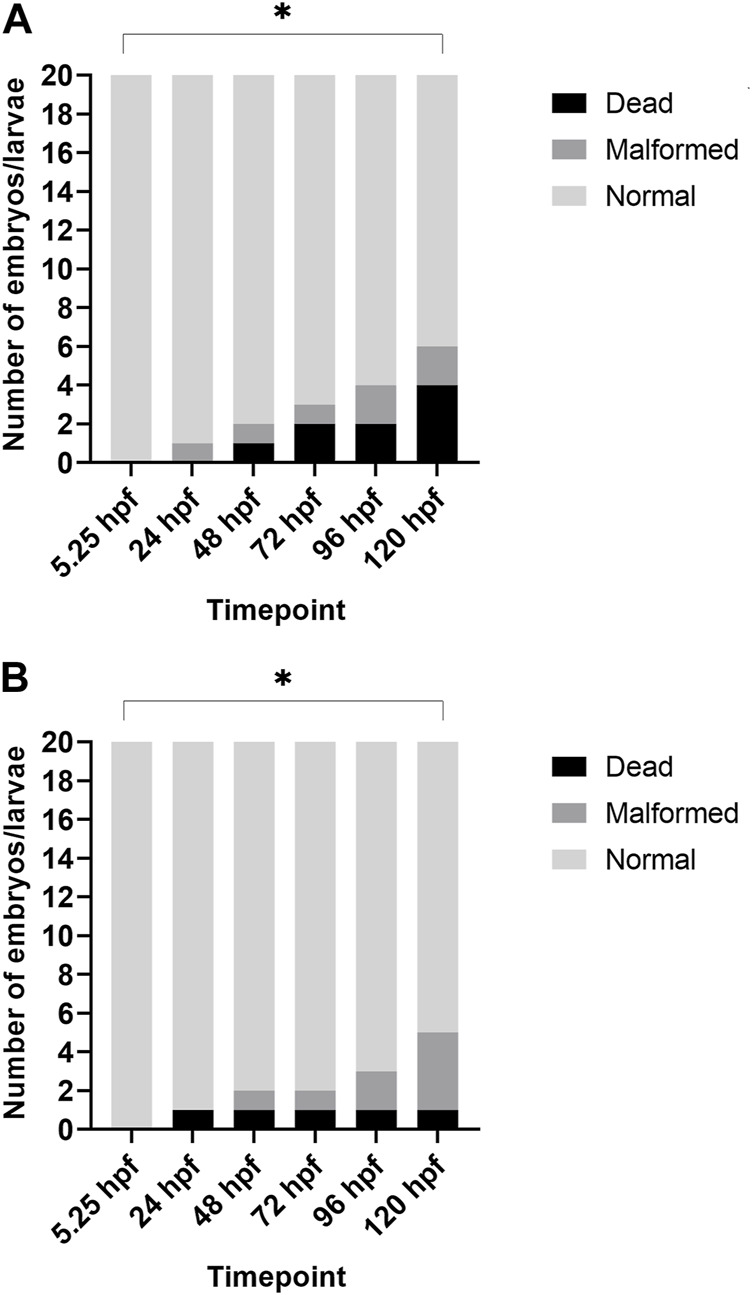
Overview of lethality and malformations for each timepoint after treatment with 2% DMSO. **(A)** depicts the results of replicate 1, and **(B)** depicts the results of replicate 2. In both replicates, the total number of dead/malformed larvae was significantly higher at 120 hpf when compared to 5.25 hpf (i.e., start of exposure). *p < 0.05.

Additionally, when looking at the 2% DMSO group at different developmental stages, we noted that the total number of malformed/dead larvae was significantly increased at 120 hpf when compared to the start of the exposure (5.25 hpf), while there was no significant increase in the total number of malformed/dead larvae at 96 hpf (see Figure 2).

### 3.2 Exposure to a Combination of DMSO and Non-teratogenic Compounds

Both replicates of the second experiment were valid, as ≥80% of all eggs were fertilized and the total number of malformed or dead larvae in the medium and solvent control groups (i.e., embryo solution and 1% DMSO) was ≤10% at the end of the experiment. The pH of all of the test solutions remained in a physiological range throughout the experiment (i.e., pH 7.60 ± 0.20) (data not shown). For both replicates, no statistical differences were observed between any of the test groups and the control groups (see Table 4 and Table 5).

**TABLE 4:** Overview of lethality and malformations in the test groups at 120 hpf in replicate 1. The ratio of affected larvae/total number of larvae is shown for each parameter and each group. For all parameters, except for *coagulation/lethality* and *total ≥ 1 malformations (incl. dead),* this total number of larvae consisted only of larvae that were alive.

Parameter	Medium control	1% DMSO (SC)	AA (Esol)	AA (1%DMSO)	HCT (1%DMSO)
Coagulation/lethality	0/20	0/20	0/20	0/20	0/20
Tot. ≥1 malf. (incl. dead)	2/20	0/20	1/20	3/20	2/20
Tot. ≥1 malf. (excl. dead)	2/20	0/20	1/20	3/20	2/20
BP indistinguishable	0/20	0/20	0/20	0/20	0/20
BP unrecognizable	0/20	0/20	0/20	0/20	2/20
No hatching	0/20	0/20	0/20	0/20	2/20
Elbow tail	0/20	0/20	0/20	1/20	2/20
Curved tail	1/20	0/20	1/20	2/20	1/20
Tissue deviation tail	1/20	0/20	1/20	1/20	2/20
Edema head	0/20	0/20	0/20	0/20	0/20
Edema pericard	0/20	0/20	1/20	1/20	2/20
Edema yolk	0/20	0/20	0/20	1/20	0/20
Edema yolk ext./tail	0/20	0/20	0/20	0/20	0/20
BA tail	0/20	0/20	0/20	1/20	0/20
BA head	0/20	0/20	0/20	0/20	0/20
BA heart	0/20	0/20	0/20	0/20	0/20
BA yolk	0/20	0/20	0/20	0/20	0/20
BA yolk extension	0/20	0/20	0/20	0/20	0/20
Missing fin left	0/20	0/20	0/20	0/20	0/20
Missing fin right	0/20	0/20	0/20	0/20	0/20
Curved fin left	0/20	0/20	0/20	0/20	0/20
Curved fin right	0/20	0/20	0/20	0/20	0/20
Malformation yolk	0/20	0/20	0/20	1/20	2/20
Malformation heart	0/20	0/20	0/20	0/20	0/20
No BC in tail	0/20	0/20	0/20	1/20	2/20
Disturbed BC in tail	0/20	0/20	0/20	0/20	0/20
Heartbeat absent	0/20	0/20	0/20	0/20	0/20
Deviating shape of head	0/20	0/20	0/20	0/20	2/20
Deviation ear	0/20	0/20	0/20	0/20	2/20
Deviation mouth	0/20	0/20	0/20	0/20	2/20
Deviation eye	0/20	0/20	1/20	0/20	2/20
Deviating pigmentation	0/20	0/20	0/20	0/20	0/20
Non-detachment tail	0/20	0/20	0/20	0/20	0/20

BA: blood accumulation, BC: blood circulation, BP: body parts, Tot. ≥1 malf (excl. dead): total number of embryos/larvae that were alive and had at least one malformation. Tot. ≥1 malf (incl. dead): total number of embryos/larvae that had at least one malformation or were dead.

**TABLE 5:** Overview of lethality and malformations in the test groups at 120 hpf in replicate 2. The ratio of affected larvae/total number of larvae is shown for each parameter and each group. For all parameters, except for *coagulation/lethality* and *total ≥ 1 malformations (incl. dead)*, this total number of larvae consisted only of larvae that were alive.

Parameter	Medium control	1% DMSO (SC)	AA (Esol)	AA (1%DMSO)	HCT (1%DMSO)
Coagulation/lethality	0/20	2/20	0/20	0/20	0/20
Tot. ≥1 malf. (incl. dead)	2/20	2/20	1/20	3/20	1/20
Tot. ≥1 malf. (excl. dead)	2/20	0/18	1/20	3/20	1/20
BP indistinguishable	0/20	0/18	0/20	0/20	0/20
BP unrecognizable	0/20	0/18	0/20	0/20	0/20
No hatching	0/20	0/18	0/20	0/20	0/20
Elbow tail	0/20	0/18	0/20	0/20	0/20
Curved tail	0/20	0/18	0/20	0/20	0/20
Tissue deviation tail	2/20	0/18	1/20	3/20	1/20
Edema head	0/20	0/18	0/20	0/20	0/20
Edema pericard	0/20	0/18	0/20	0/20	0/20
Edema yolk	0/20	0/18	0/20	0/20	0/20
Edema yolk ext./tail	0/20	0/18	0/20	0/20	0/20
BA tail	0/20	0/18	0/20	0/20	0/20
BA head	0/20	0/18	0/20	0/20	0/20
BA heart	0/20	0/18	0/20	0/20	0/20
BA yolk	0/20	0/18	0/20	0/20	0/20
BA yolk extension	0/20	0/18	0/20	0/20	0/20
Missing fin left	0/20	0/18	0/20	0/20	0/20
Missing fin right	0/20	0/18	0/20	0/20	0/20
Curved fin left	0/20	0/18	0/20	0/20	0/20
Curved fin right	0/20	0/18	0/20	0/20	0/20
Malformation yolk	0/20	0/18	0/20	0/20	0/20
Malformation heart	0/20	0/18	0/20	0/20	0/20
No BC in tail	0/20	0/18	0/20	0/20	0/20
Disturbed BC in tail	0/20	0/18	0/20	0/20	0/20
Heartbeat absent	0/20	0/18	0/20	0/20	0/20
Deviating shape of head	0/20	0/18	0/20	0/20	0/20
Deviation ear	0/20	0/18	0/20	0/20	0/20
Deviation mouth	0/20	0/18	0/20	0/20	0/20
Deviation eye	0/20	0/18	0/20	0/20	0/20
Deviating pigmentation	0/20	0/18	0/20	0/20	0/20
Non-detachment tail	0/20	0/18	0/20	0/20	0/20

BA: blood accumulation, BC: blood circulation, BP: body parts, Tot. ≥1 malf (excl. dead): total number of embryos/larvae that were alive and had at least one malformation. Tot. ≥1 malf (incl. dead): total number of embryos/larvae that had at least one malformation or were dead.

In both replicates, there were a few more malformed larvae at 120 hpf after treatment with a combination of 100 µM of AA and 1% DMSO (3/20 or 15%), than after treatment with 100 µM AA alone (1/20 or 5%) or 1% DMSO alone (0/20 or 0% in replicate 1 and 2/20 or 10% in replicate 2). However, no statistical differences were noted. In both AA treated groups of the second replicate, only tissue deviations of the tail were observed (see Table 5). In the first replicate, a wider variety of malformations was observed (see Table 4).

For the second compound, HCT, similar results were obtained. There were no significant differences between the group treated with 1,000 µM of HCT with 1% DMSO and the solvent control (see Table 4 and Table 5). In the first replicate, there were only a few more malformations in the group treated with the combination of DMSO and HCT than in the solvent control. However, when the group treated with a combination of DMSO and HCT was compared with the medium control group, the total number of embryos/larvae that had at least one malformation was the same (i.e., 2/20 or 10%) (see Table 4). In the second replicate, the total number of embryos/larvae that had at least one malformation or were dead was even less than in the solvent control and medium control groups (see Table 5).

## 4 Discussion

Our study showed no statistical increase in lethality nor gross morphology malformations up to 120 hpf in all DMSO test groups when compared with the medium control group (0% DMSO). However, at 2% DMSO, more than 25% of the larvae had at least one malformation or died in both replicates and one of the replicates showed 20% dead embryos at 120 hpf. As such, 2% DMSO cannot be used as solvent control group in future experiments with our standardized ZEDTA, as the total number of malformed and/or dead larvae in the (solvent) control group needs to be ≤ 10%, in order to have a valid experiment. Furthermore, when comparing the number of malformed and dead embryos in the 2% DMSO group at the start of exposure (5.25 hpf) with the number of malformed and dead embryos at the end of exposure (120 hpf), a significant increase was noted, which was absent at 96 hpf. These data show that extending the exposure in the ZEDTA to 120 hpf instead of 96 hpf makes the assay more sensitive. Also studies using shorter exposure periods indicate that extending the exposure window until 120 hf might be of added value (Maes et al., 2012; Huang et al., 2018). Huang et al. (2018) showed that zebrafish mortality increased and LC50 values decreased in later developmental stages (Huang et al., 2018) and Maes et al. (2012) also found that later developmental stages were more sensitive than the earlier stages when exposing them for a 24 h period (Maes et al., 2012). In many other studies, the exposure period ends at 96 hpf (Hallare et al., 2004, 2006; Huang et al., 2018; Kim and Lee, 2021), but based upon the data above we recommend extending the exposure period in the ZEDTA to 120 hpf in any further experiments.

When looking more into detail to the malformations at 2% DMSO, several types of edema were observed, which was in line with other studies (Hallare et al., 2006; Xiong et al., 2017). However, tissue deviation of the tail or/and the body (i.e. a collective term for all abnormalities that are visible in the tissue of the tail or/and body) showed to be the most prominent malformations in our study, and manifested itself as cell death in these areas. These malformations have not been reported in any of the above studies, but they may have been missed, as this parameter was not included in their list of endpoints. Regarding lethality, Xiong et al. (2017) also reported an increase at 2% DMSO, as in our study, but at a later developmental stage, i.e. 7 dpf (days post-fertilization) (Xiong et al., 2017). Hallare et al. (2006) found no effect on survival when exposing embryos/larvae to up to 2% (Hallare et al., 2006), but their exposure period was 24 h shorter (i.e. up to 96 hpf) than in our study, confirming again the importance of extending the exposure period to 120 hpf in the ZEDTA.

Based on the data above, 1% DMSO appears to be the maximum tolerated concentration in our standardized ZEDTA. However, as other authors showed toxic effects when combining the transitional metal vanadium with 0.1 and 0.5% DMSO (Kim and Lee, 2021), which were absent when exposing the embryos solely to vanadium or 0.1 and 0.5% DMSO, we wanted to assess whether 1% DMSO does not cause developmental toxicity when combined with non-teratogens. Kim and Lee (2021) could relate the toxic effect to a significant decrease in pH when combining vanadium with DMSO. Large pH drops, i.e. from pH ∼7 to pH ∼4, were reported (Kim and Lee, 2021) and drastic changes in pH are well-known to have a negative impact on zebrafish development (Dave, 1984; Andrade et al., 2017). In our study, combining ascorbic acid and hydrochlorothiazide with 1% DMSO did not cause any developmental toxicity and the pH of the exposure medium remained within the physiological range (i.e. pH 7.60 ± 0.20).

In conclusion, we showed that 1% of DMSO can be safely used to dissolve chemicals in the ZEDTA. However, caution is needed for compounds that, with or without DMSO, change the pH of the exposure medium. We therefore recommend to check the pH of all test solutions, and adjust them to a physiological pH when needed. Furthermore, we only assessed the maximum tolerated concentration of DMSO in zebrafish embryos for developmental toxicity. When zebrafish embryos are used for other types of toxicity or when other endpoints than gross morphology are examined (e.g. hsp70 levels and behavioural responses), the DMSO concentrations may need to be further reduced, as already reported in other studies (Hallare et al., 2004, 2006; Chen et al., 2011; Xiong et al., 2017).

## Data Availability

The original contributions presented in the study are included in the article/Supplementary Material further inquiries can be directed to the corresponding author.
